# YOLO-SEA: An Enhanced Detection Framework for Multi-Scale Maritime Targets in Complex Sea States and Adverse Weather

**DOI:** 10.3390/e27070667

**Published:** 2025-06-22

**Authors:** Hongmei Deng, Shuaiqun Wang, Xinyao Wang, Wen Zheng, Yanli Xu

**Affiliations:** 1 College of Information Engineering, Shanghai Maritime University, Shanghai 201306, China; denghongmei@stu.shmtu.edu.cn (H.D.); ylxu@shmtu.edu.cn (Y.X.); 2 Innovation Academy for Microsatellites of Chinese Academy of Sciences, Shanghai 201210, China; zhengwen@microsate.ac.cn

**Keywords:** maritime target detection, YOLOv8, deep learning, multi-target recognition

## Abstract

Maritime object detection is essential for resource monitoring, maritime defense, and public safety, yet detecting diverse targets beyond ships remains challenging. This paper presents YOLO-SEA, an efficient detection framework based on the enhanced YOLOv8 architecture. The model incorporates the SESA (SimAM-Enhanced SENetV2 Attention) module, which integrates the channel-adaptive weight adjustment of SENetV2 with the parameter-free spatial-channel modeling of SimAM to enhance feature representation. An improved BiFPN (Bidirectional Feature Pyramid Network) structure enhances multi-scale fusion, particularly for small object detection. In the post-processing stage, Soft-NMS (Soft Non-Maximum Suppression) replaces traditional NMS to reduce false suppression in dense scenes. YOLO-SEA detects eight maritime object types. Experiments show it achieves a 5.8% improvement in mAP@0.5 and 7.2% improvement in mAP@0.5:0.95 over the baseline, demonstrating enhanced accuracy and robustness in complex marine environments.

## 1. Introduction

As global attention to marine resource exploitation, environmental conservation, maritime safety, and the protection of maritime interests continues to grow, ocean monitoring and management are encountering increasingly complex challenges. Maritime environments contain a diverse range of dynamic targets—such as naval vessels, fishing boats, cargo ships, and offshore structures—characterized by significant variation in shape, scale, and motion patterns. This diversity presents substantial difficulties for accurate detection and localization. Robust and efficient detection of multi-class maritime targets is not only crucial for enhancing global maritime traffic safety, emergency response, and resource management but also provides essential technological support for national and international marine governance and strategic decision-making [1].

The dynamic and unpredictable nature of the marine environment complicates detection tasks. Factors like wave motion, variable lighting, sea fog, and adverse weather degrade visual quality, reducing the reliability of traditional detection algorithms [2]. Additionally, differences between nearshore and offshore scenes, static and moving targets, and imbalanced target classes require more adaptable detection solutions [3]. Developing advanced models for accurate multi-scale, multi-class, and multi-object detection in complex marine conditions is therefore crucial. Advancements in this area will accelerate smart maritime technologies and autonomous navigation while supporting sustainable ocean resource use and enhancing global maritime security.

Conventional techniques employ convolutional templates to extract feature information from images, utilizing methods such as HOG [4] (Histogram of Oriented Gradients) and SIFT [5] (Scale-Invariant Feature Transform). These features are subsequently integrated with classifiers—notably, Support Vector Machine (SVM) [6]—to facilitate target classification and recognition. Building on this, studies have combined HOG [7] features with SVM classifiers for marine oil spill detection, enhancing low-altitude monitoring accuracy and enabling all-weather offshore surveillance. Other works have developed SVM-based frameworks [8] to extract key ship defect features for effective prediction of vessel detention events.

Recent advances in computer vision, image analysis, and pattern recognition have been driven by significant progress in artificial intelligence [9], especially in computational power, large-scale data collection, and deep learning optimization. These advancements have led to improved accuracy and efficiency in maritime target detection. Deep learning-based recognition technologies, particularly convolutional neural network models like SSD (Single-Shot MultiBox Detector), Faster R-CNN (Faster Region-based Convolutional Neural Network), and YOLO (You Only Look Once) have become the standard for target detection due to their superior capabilities and wide applicability. To meet the real-time recognition demands of complex marine environments, extensive experiments have been conducted using these models to continuously improve detection accuracy, computational efficiency, and generalization. A convolutional neural network model integrating multiple strategies [10] was developed for ship target detection. Experimental results show that the model outperforms mainstream methods such as SSD and Faster R-CNN in detection accuracy. Cross-polarized C-band SAR (Synthetic Aperture Radar) images [11] have been utilized to implement Faster R-CNN, RetinaNet, and single-stage detectors on various ResNet backbones, enabling performance and adaptability comparisons across detection frameworks. A Faster R-CNN-based [12] approach integrates dilated and group convolutions into a multi-scale feature extraction module and optimizes the classification strategy, improving the robustness and accuracy of unmanned surface-vessel obstacle detection. To address reduced detection accuracy under sea-fog conditions, a method combining dehazing preprocessing with an SSD-based detection model [13] has been proposed, enhancing maritime vessel detection in adverse weather environments.

Convolutional neural networks such as the YOLO [14] model serve as the basis for deep learning algorithms. The YOLOv2 network, combined with the SELU (Scaled Exponential Linear Unit) activation function [15], achieves excellent detection accuracy and speed for small ship detection. An improved YOLOv3-Tiny detection method [16] was proposed based on a feature pyramid network, enhancing detection accuracy through feature fusion. This method integrates the second scale output layer with the fourth DBL (Conv + BatchNorm + LeakyReLU) output and introduces a 52 × 52 scale output, improving accuracy while maintaining detection speed, with results validated on a self-built dataset. An improved YOLOv3 model [17] introduces new anchor-setting methods and a cross-PANet structure with focal loss, boosting sea target detection accuracy. However, its performance still depends on anchor settings, limiting generalization. In the feature fusion module of YOLOv4, a convolutional attention module [18] is introduced to weight channel and spatial features, significantly improving maritime target detection accuracy. An improved YOLOv5 model incorporates weighted clustering for data processing and introduces a BN (Batch Normalization) scaling factor for lightweight implementation [19], boosting real-time ship detection performance. Despite its high accuracy and robustness, the recall rate for small targets remains to be improved. An improved YOLOv5 model [20] incorporates weighted clustering for data processing and introduces a BN scaling factor for lightweight implementation, boosting real-time ship detection performance. Despite its high accuracy and robustness, the recall rate for small targets remains to be improved.

To improve target detection accuracy and generalization capacity in complicated maritime situations, this work suggests an enhanced YOLOv8 algorithm for multi-category, multi-target, and multi-scale high-precision detection at sea. The following are the primary contributions:

1. The dataset encompasses a range of marine objects, including common ships, buoys, lighthouses, and aircraft operating on the sea surface. It features eight distinct target categories characterized by significant diversity and variety. This approach aligns more closely with the complexity of the actual offshore maritime environment compared to a traditional single-ship dataset.
2. The backbone network integrates the SESA (SimAM-Enhanced SENetV2 Attention) fusion attention module, which combines the parameter-free feature modeling mechanism of the Simple, Parameter-Free Attention Module (SimAM) with the channel-adaptive weight adjustment of Squeeze-and-Excitation Network Version 2 (SENetV2) across the three dimensions of channel, height, and width. It also examines the spatial distribution of features and the significance of the channel. This synergistic mechanism effectively captures key features of multi-scale and multi-category targets in complex maritime scenes, enhancing their differentiation and expression capabilities. This leads to increased detection accuracy and robustness, along with a more thorough and reliable target sensing capability.
3. The neck section presents an enhanced fusion model utilizing a Bidirectional Feature Pyramid Network (BiFPN). This model facilitates information interoperability across feature levels through the weighted fusion of diverse scale feature information via bidirectional cross-scale connections. It strengthens the integration of high-level semantics with low-level detail features, thereby significantly improving the detection accuracy of multi-scale targets in complex environments.
4. Soft Non-Maximum Suppression (Soft-NMS) utilizes weight attenuation to dynamically modify the confidence levels of candidate boxes based on their overlap rather than outright rejection, thereby preserving more valid information. This substantially enhances the average accuracy of current object detection algorithms in the detection of multiple overlapping objects.
5. A test dataset was created to assess the detection model’s performance under various severe weather conditions, including three scenarios: rainy weather, hazy weather, and low-light environments.

The remainder of this paper is organized as follows: Section 2 provides a comprehensive description of the proposed methodological improvements. Section 3 subsequently presents extensive experimental validation, including ablation studies and generalization tests, along with comparative performance analyses against existing approaches. Finally, Section 4 concludes the study by summarizing key findings and outlining potential directions for future research in this domain.

## 2. YOLO-SEA Model and Optimization

### 2.1. YOLOv8 Network

YOLOv8 [21] comprises three components: the backbone network, the feature fusion network, and the detection head, which collaboratively function to facilitate efficient target detection. The backbone network comprises CBL, C2f, and SPPF modules. CBL denotes the traditional feature extraction process, which includes convolution, Batch Normalization (BN), and an activation function. C2f serves as the primary feature learning module, extracting and integrating features via a residual module to improve feature representation capabilities. The Spatial Pyramid Pooling Fast (SPPF) module integrates local and global feature information via tandem max-pooling computation, thereby improving the capability of feature representation. The feature fusion network utilizes the Path Aggregation Network (PAN) structure, which enhances the fusion capability of features from objects at varying scales, thereby improving detection performance for multi-scale targets. The detection head analyzes the target category and location of extracted features while simultaneously screening positive and negative samples and computing the loss. YOLOv8 employs the task-aligned assigner method to differentiate between positive and negative samples. The loss function comprises two components: the classification loss utilizes Binary Cross Entropy (BCE), while the regression loss integrates distributed focal loss. Distributed Focal loss and CIOU are employed to achieve precise target localization and classification.

### 2.2. YOLO-SEA Algorithm

**Figure 1:**
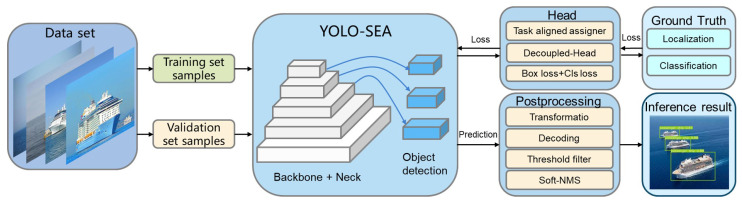
Workflow diagram of YOLO-SEA.

**Figure 2:**
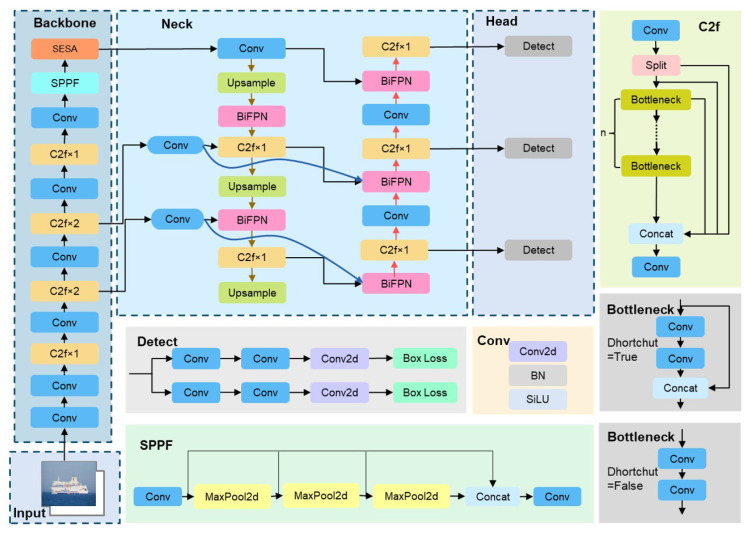
Network architecture diagram of YOLO-SEA.

The YOLO-SEA model is derived from the YOLOv8 framework, optimized for detection tasks involving multiple categories, targets, and scales in maritime environments. Figure 1 illustrates the workflow of YOLO-SEA. The input images are standardized to a resolution of 640 × 640 to meet the model’s processing specifications. The backbone network integrates C2f, Conv, and SESA fusion attention mechanisms to facilitate efficient feature extraction. The SESA fusion attention mechanism integrates the channel-adaptive weight adjustment of SENetV2 with the parameter-free spatial-channel modeling of SimAM, enhancing attention across both spatial and channel dimensions, thereby enhancing the representation of critical features. The SESA fusion attention mechanism effectively captures unique features of targets across various scales, thereby improving the network’s adaptability to complex scenes, particularly in multi-scale and multi-class target detection tasks. Category target detection tasks exhibit superior performance. The neck network utilizes an optimized BiFPN structure to enhance cross-layer feature fusion and improve the detection performance of multi-scale targets. Soft-NMS is implemented in the final detection stage, substituting traditional non-maximum suppression (NMS) with confidence decay. This approach effectively decreases false and missed detection rates for dense targets and exhibits superior detection performance in complex maritime environments. Figure 2 illustrates the network architecture of YOLO-SEA.

#### 2.2.1. SESA Fusion Attention Mechanism

This paper presents the design and integration of a fusion attention module, SESA (SimAM-Enhanced SENetV2 Attention), into the backbone network. The module optimizes channel importance and spatial distribution features synergistically by integrating the channel-adaptive weight adjustment mechanism of Squeeze-and-Excitation Network Version 2 (SENetV2) with the parameter-free feature modeling method of the Simple Attention Module (SimAM) across the three dimensions of channel and spatial dimensions (height and width). The SENetV2 module adjusts the weight of each channel dynamically through global information compression and inter-channel relationship modeling to emphasize important features. In contrast, SimAM employs a parameter-independent approach to enhance feature expression by capturing correlations between local space and channels.

This fusion mechanism enhances feature expression and differentiation capabilities for multi-scale and multi-category targets. It demonstrates improved detection accuracy and robustness, particularly in complex maritime scenes with dense and scale-differentiated targets. The SESA fusion attention mechanism enhances fine-grained feature expression in target detection by concurrently integrating attention mechanisms across multiple dimensions. This approach improves the network’s focus on critical features, thereby increasing the model’s perceptiveness and adaptability in varied and complex environments. The two modules are presented individually in the subsequent sections.

- SENetV2 Module

**Figure 3:**
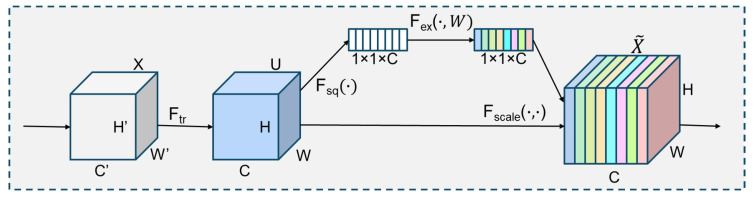
Extrusion-Excitation Module for SENetV2 Modules.

SENetV2 [22] notably improves the feature representation capacity of the network through enhancements to the Squeeze-and-Excite (SE) module. This module, grounded in the traditional SE structure, incorporates a multi-branch, fully connected layer to enhance the efficiency of global information capture and optimize feature weight distribution. The proposed Squeeze Aggregation Excitation (SaE) module improves feature transfer efficiency and enhances feature expression capability by increasing the inter-layer basis and reducing parameter redundancy, all while maintaining a lightweight design to ensure high model efficiency, as shown in Figure 3. The SaE [22] module normalizes the outputs of the aggregated fully connected layers during the excitation phase and employs a lightweight gating mechanism to dynamically adjust inter-channel dependencies, enhancing the prominence of key features. This optimization strategy enhances the model’s feature extraction capabilities in complex scenes and improves recognition robustness for targets of varying scales, thereby establishing a strong foundation for accurate target detection.

**Figure 4:**
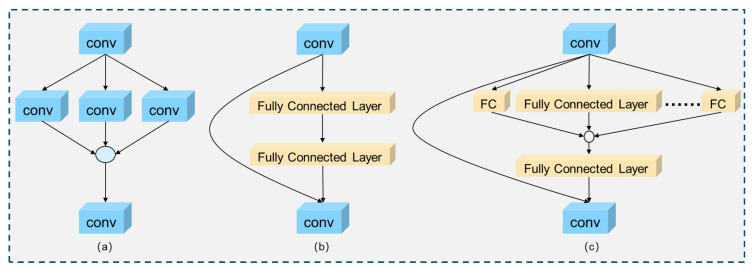
(**a**) ResNeXt module. (**b**) SENet module. (**c**) SENetV2 module.

ResNeXt [23] enhances residual modules by incorporating a novel dimension of “cardinality”, which decreases theoretical complexity and enhances performance relative to conventional partial architectures. The Wide Residual Network [24] (WRN) enhances performance by increasing the width of the top layer compared to traditional ResNet architectures. Figure 4a illustrates the internal functionality of ResNeXt, whereas SENet [25] enhances model performance by integrating channel representations via novel squeezing and excitation modules. The highway network implements a gating mechanism to control the function of connections. Figure 4b illustrates the internal functions of SENet.

SENetV2 [22] enhances the modeling capability of the channel attention mechanism through the implementation of a multi-branch, fully connected (FC) layer structure. Figure 4c illustrates that the module initially compresses the feature graph via global average pooling to obtain global information. The parallel fully connected layer is employed to identify the nonlinear relationships between channels to generate channel weights. The weights derived from feature scaling operations are applied to the original feature maps to enhance key channel features adaptively. This process enhances the network’s ability in feature extraction, allowing the model to concentrate more accurately on critical channel information, thereby optimizing feature representation and improving target recognition in complex scenes.

SENetV2 is utilized alongside ResNet to incorporate shortcuts that can circumvent one or more layers integrated via these shortcuts. The input in the basic version of the residual module is represented as x. The operations that modify the input, such as batch normalization and dropout, are represented by F(). The residual module is mathematically expressed as follows:

(1) $$\begin{array}{c} \mathrm{ResNet}=x+F(x) \end{array}$$

In the aggregated residual module, branch convolution allows for the direct concatenation of inputs without modifications. The mathematical representation of the ResNeXt module is presented below:

(2) Resnet=x+∑F(x)

The SENetV2 module integrates squeezing and excitation techniques. The squeezing process compresses the input via a fully connected (FC) layer, which initially directs the output of the sparse layer to a global average pooling layer that produces channel-dependent features. The features are input into the fully connected layer and undergo size reduction processing. Conversely, the inspired component reverts the input to its original state by utilizing the fully connected layer without any size reduction. The FC layer is inherited during the scaling operation, where the output is multiplied by the channel direction of the feature map and subsequently resized to match the original shape. The formula for the extrusion and excitation operations of the module is expressed as follows:

(3) $$\begin{array}{c} \mathrm{SEnet}=x+F\left(x\cdot \mathrm{Ex}(\mathrm{Sq}(x))\right) \end{array}$$

The Sq function denotes the squeeze operation and includes a fully connected layer with a reduced size indicated by *r*. After the Sq operation, the Ex operation is executed, denoting the excitation operation.

The squeezing operation involves a size-reduced fully connected (FC) layer that processes the output from global average pooling. This transformation preserves essential features, facilitating their efficient propagation through the modules and thereby enhancing the network’s expressive power. The aggregation layers undergo a squeezing operation before being transmitted to the fully connected layer. The output of the fully connected layer is subsequently multiplied element-wise with the module inputs to achieve dimensionality recovery. The sequence of operations is outlined as follows:

(4) $$\begin{array}{c} \mathrm{SEnetV}2=x+F\left(x\cdot \mathrm{Ex}\left(\sum \mathrm{Sq}(x)\right)\right) \end{array}$$

B. SimAM Attention Mechanism

The SimAM attention mechanism, inspired by human neuronal activity, replicates neuronal activation patterns and effectively suppresses the activity of neighboring neurons that lack sufficient information. SimAM effectively identifies and reinforces features that contain significant information by defining a specific energy function for each neuron. Incorporating this biological principle into machine learning, SimAM systematically learns similarity information among targets, computes similarity metrics between features, and allocates greater weights to significant features. SimAM significantly enhances model performance in fine-grained tasks. The central concept revolves around the variability of feature contributions, where distinct features possess varying significance for the task. SimAM directs the model’s attention toward the most informative elements by emphasizing salient features, thereby enhancing the efficiency and accuracy of target detection [26]. An energy function is defined for each neuron, as presented in Equation (5).

(5) $$e_{t}\left(w_{t},b_{t},y,x_{i}\right)={\left(y_{t}-\hat{t}\right)}^{2}+\frac{1}{M-1}\sum_{i=1}^{M-1}{\left(y_{0}-{\hat{x}}_{i}\right)}^{2}$$

The *M* variable denotes the number of neurons in the preceding equation, where $\hat{t}=w_{t}t+b_{t}$ and ${\hat{x}}_{i}=w_{t}x_{i}+b_{t}$ represent the linear transformations of the target neuron and the other neurons within the input feature map of the same channel, respectively [27]. The computation is simplified by assigning binary labels to scalars $y_{t}$ and $y_{0}$, while regularization term λ is incorporated through a reconfiguration of the energy equation, resulting in Equation (6).

(6) $$\begin{array}{ccc} e_{t}\left(w_{t},b_{t},y,x_{i}\right) & = & \frac{1}{M-1}\sum_{i=1}^{M-1}{\left(-1-(w_{t}x_{i}+b_{t})\right)}^{2}\\ & & +{\left(-1-(w_{t}t+b_{t})\right)}^{2}+\lambda w_{t}^{2} \end{array}$$

The solution to the aforementioned equation yields a weight of $w_{t}$ and a deviation of $b_{t}$.

(7) $$w_{t}=\frac{-2(t-\mu_{t})}{{\left(t-\mu_{t}\right)}^{2}+2\sigma_{t}^{2}+2\lambda }$$

(8) $$b_{t}=-\frac{1}{2}\left(t+\mu_{t}\right)w_{t}$$

$\mu_{t}$ and σ indicate the mean variance of each neuron within that channel:

(9) $$e_{t}^{*}=\frac{4(\sigma^{2}+\lambda )}{{\left(t-\hat{\mu }\right)}^{2}+2\sigma^{2}+2\lambda }$$

The equation indicates that a neuron’s saliency is inversely related to its energy value ($e_{t}^{*}$). A significant increase in saliency occurs when $e_{t}^{*}$ is reduced. A lower energy value signifies greater differentiation of the neuron from adjacent neurons, thereby enhancing its prominence in feature representation. Applying Equation (10) [28] to a deep neural network yields the following final formula:

(10) $$\tilde{X}=\mathrm{Sigmoid}\left(\frac{1}{E}\right)X$$

**Figure 5:**
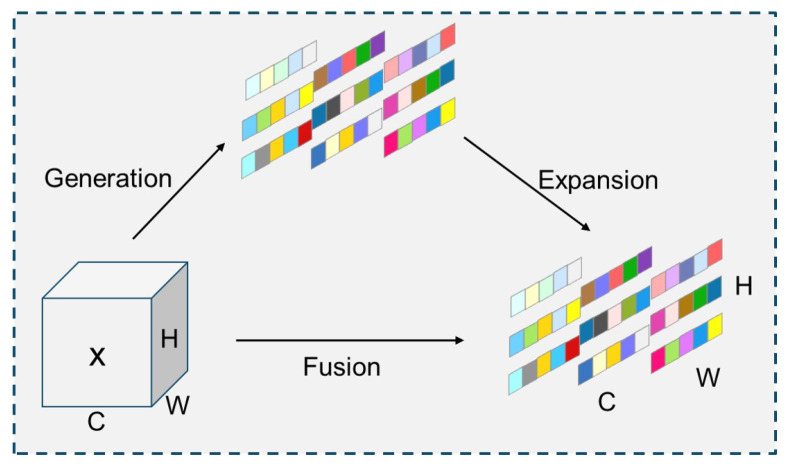
SimAM attention module structure.

SimAM computes feature significance in spatial and channel dimensions without parameters, effectively capturing essential information while minimizing redundant features. This enhances the model’s focus on the target core region and improves detection accuracy. Figure 5 illustrates that the SimAM module quantifies feature significance using the energy function, which is integrated with the Sigmoid function to assign weights to the features, thereby enabling adaptive enhancement of significant features. The design demonstrates computational efficiency and avoids the introduction of additional parameters, thereby enhancing performance while preserving the model’s lightweight characteristics [29,30,31].

#### 2.2.2. Bidirectional Feature Pyramid Network (BiFPN)

The feature fusion network of YOLOv8 utilizes the Path Aggregation Network [32] (PANet), which addresses the accurate localization of target locations through extended pathways from bottom to top. The network is characterized by various forms of connections, including top-down, top-to-bottom, bottom-up, bottom-to-top, and lateral interactions. High-level features typically encompass extensive high-dimensional semantic information, enhancing object classification tasks yet potentially compromising localization accuracy. Conversely, low-level features yield more precise object localization but are deficient in semantic richness. The integration of high-level semantic features with low-level localization information through a feature fusion network enhances the expressiveness of all features, reduces the distance of information transfer [33], and improves the performance of the overall feature hierarchy.

**Figure 6:**
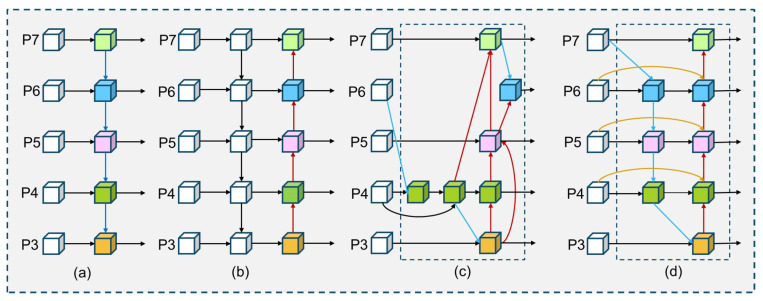
Differences between different characterized pyramid networks. (**a**) FPN. (**b**) PANet. (**c**) NAS-FPN. (**d**) BiFPN.

The Bidirectional Feature Pyramid Network (BiFPN) was introduced by Efficient-Det [34] to enhance feature fusion and accuracy while maintaining low computational costs. Figure 6d illustrates that BiFPN, which is founded in PANet, achieves cross-scale flow and fusion of feature information through the incorporation of jump connections between input and output nodes within the same layer. Moreover, BiFPN introduces a rapid normalized fusion algorithm that allocates varying weights to each feature layer based on its significance for efficient fusion. This method enhances the network’s focus on critical feature layers while simultaneously minimizing node connections in less essential layers to optimize overall performance.

The fast normalized fusion algorithm expressed is as follows:

(11) $$p_{i}^{\mathrm{td}}=\mathrm{Conv}\left(\frac{w_{1}\cdot p_{i}^{\mathrm{in}}+w_{2}\cdot \mathrm{Resize}\left(p_{i+1}^{\mathrm{in}}\right)}{w_{1}+w_{2}+\varepsilon }\right)$$

(12) $$p_{i}^{\mathrm{out}}=\mathrm{Conv}\left(\frac{w_{1}^{\prime }\cdot p_{i}^{\mathrm{in}}+w_{2}^{\prime }\cdot p_{i}^{\mathrm{td}}+w_{3}^{\prime }\cdot \mathrm{Resize}\left(p_{i-1}^{\mathrm{out}}\right)}{w_{1}^{\prime }+w_{2}^{\prime }+w_{3}^{\prime }+\varepsilon }\right)$$

Let $p_{i}^{in}$ represent the feature map of the ith layer, $p_{i}^{td}$ denote the intermediate feature map of the ith layer, and $p_{i}^{out}$ indicate the output map of the ith layer, where λ=0.0001.

This paper presents an enhanced feature pyramid structure derived from BiFPN, incorporating five feature extraction layers: two for small targets, two for medium targets, and one for large targets. The design aligns with the characteristics of objects in maritime scenes, retaining only one layer due to the significant proportion of small and medium targets, while larger targets are more readily detectable. The original BiFPN employs a rapid normalized fusion algorithm for feature fusion; however, the complex maritime background can significantly disrupt the underlying features, thereby impairing the detection of small targets. We optimize the BiFPN structure to enhance its adaptability to the features of maritime objects. The detection accuracy is enhanced, and improved multi-scale feature extraction is achieved without increasing the number of model parameters and Floating Point Operations Per second (FLOPs).

#### 2.2.3. Soft-NMS Module

This paper presents the integration of the Soft-NMS algorithm with the Gaussian reset method as a substitute for the conventional NMS algorithm within the YOLOv8 framework. Soft-NMS necessitates minimal adjustment of the conventional NMS method and does not introduce extra parameters, thereby preserving the algorithm’s implementation simplicity. Its complexity remains comparable to that of traditional NMS while avoiding additional computational demands.

**Figure 7:**
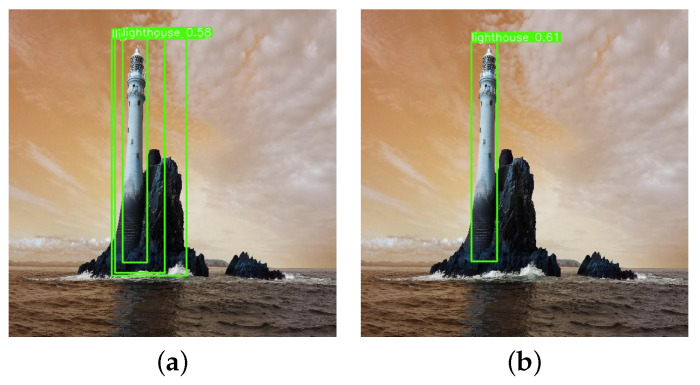
Comparison before and after applying Soft-NMS. (**a**) YOLOv8. (**b**) YOLOv8 + Soft-NMS.

Soft-NMS [35] can be integrated into the existing target detection pipeline without requiring additional model training, thereby enhancing its practicality. The introduction of a confidence decay mechanism in Soft-NMS addresses the target leakage issue arising from overlapping proposals, thereby improving the robustness and accuracy of target detection systems in complex scenarios, particularly in UAV imagery, where target occlusion and overlapping are significant challenges. Traditional NMS operates based on the IoU (intersection over union), which can inadvertently eliminate significant targets. In contrast, Soft-NMS preserves the information of high-confidence frames and enhances the localization accuracy of bounding boxes by dynamically modifying the weight of the bounding box about the confidence level and the extent of overlap. Figure 7 provides a clear visual comparison of the detection results using both methods.

The integration of the Gaussian reset method with Soft-NMS enhances its resilience to the suppression of overlapping regions, thereby improving its effectiveness in addressing occlusion issues in complex scenes and significantly decreasing the likelihood of false and missed detections in these areas. The mathematical expression is as follows:

(13) $$s_{i}=\left\{\begin{array}{cc} s_{i}, & \mathrm{IoU}\left(M,b_{i}\right)<N_{t}\\ s_{i}\left(1-\mathrm{IoU}\left(M,b_{i}\right)\right), & \mathrm{IoU}\left(M,b_{i}\right)\geq N_{t} \end{array}\right.$$

The iou(.) expression quantifies the intersection ratio within the *i*-th candidate box. *M* and Nt represent the established limit, while $s_{i}$ indicates the outcome of the *i*-th candidate box. *M* and $b_{i}$ represent the coordinates of the candidate box associated with the maximal score and the coordinates of the *i*-th candidate box, respectively.

## 3. Experiments

### 3.1. Experimental Environment and Dataset

#### 3.1.1. Experimental Environment

The experimental environment utilizes the Ubuntu 18.04.2 LTS operating system and a GPU network featuring an NVIDIA GeForce RTX 4090 Ti, which has 10 GB of video memory for experimental operations. The software environment consists of PyTorch v2.4.1 operating on the Ubuntu 20.04 system. The input image is standardized to 640 × 640, and the number of epochs is established at 300 during the training process. This study employs stochastic gradient descent (SGD) with a learning rate of 0.01 and a momentum of 0.01 for network optimization. This paper employs default parameters for training during the experiments.

#### 3.1.2. Dataset Acquisition

This study verifies the performance of YOLO-SEA using a dataset of 1934 images sourced from the Roboflow website. The dataset encompasses eight categories: aircraft carriers, buoys, freighters, fishing boats, helicopters, lighthouses, passenger ships, and warships. These categories are selected based on their typicality in maritime environments, visual distinguishability, and the availability of sufficient annotated samples, ensuring a balanced class distribution conducive to effective model training and evaluation. The images are randomly partitioned into training, validation, and test sets in a 7:2:1 ratio. Labeling is conducted utilizing LabelMe. Table 1 presents the details of the dataset.

### 3.2. Evaluation Models

The YOLOv8 model comprises five versions: YOLOv8n, YOLOv8s, YOLOv8m, YOLOv8l, and YOLOv8x. The network characteristics remain consistent; however, there are variations in network depth and width. Experiments could theoretically be conducted using any of these versions. This study primarily focuses on the smallest version of the model, YOLOv8n, due to limitations in computational resources.

This paper evaluates the detection capability of YOLO-SEA using model size, detection speed per image, precision (P), recall (R), F1 score, mAP@0.5, mAP@0.5:0.95, and additional metrics. Evaluation metrics are defined based on specific parameters: true positives (TPs) refer to instances where the prediction accurately identifies a positive class, false positives (FPs) refer to instances where the prediction inaccurately identifies a positive class, and false negatives (FNs) refer to instances where the prediction inaccurately identifies a negative class. The ratio of their intersection to concatenation, known as the Intersection over Union (IoU), reflects the percentage of overlap between the bounding box and the true box.

#### 3.2.1. Precision and Recall

Equation (14) illustrates that precision [36] is determined by the ratio of positively predicted samples to the total number of observed samples.

(14) $$\mathrm{Precision}=\frac{\mathrm{TP}}{\mathrm{TP}+\mathrm{FP}}$$

The recall rate is defined as the proportion of positive predictor samples accurately identified by the model relative to the total number of positive samples available. Equation (15) calculates the recall rate.

(15) $$\mathrm{Recall}=\frac{\mathrm{TP}}{\mathrm{TP}+\mathrm{FN}}$$

Precision–recall curves (P-R curves) serve as effective representations of visualization model performance. The study by Boyd et al. [37] presented recall on the x-axis and precision on the y-axis in its plots.

#### 3.2.2. F1 Score

The F1 score [36] provides an accurate evaluation of algorithm performance by considering both precision and recall. Equation (16) illustrates the calculation of the F1 score.

(16) $$F_{1}=\frac{2\cdot \mathrm{Precision}\cdot \mathrm{Recall}}{\mathrm{Precision}+\mathrm{Recall}}$$

In image recognition tasks, the F1 score serves as a metric for assessing an algorithm’s capability in target detection. This paper utilizes the F1 score as the primary metric for assessing model efficacy.

#### 3.2.3. Average Precision mAP

The area beneath the precision–recall curve is equivalent to the average precision (AP) and can be computed using the subsequent equation.

(17) AP=∫P(R)dR

Mean accuracy precision [36] (mAP) serves as a comprehensive metric for evaluating the detection performance of a model, derived from a weighted average of the average precision (AP) values across each sample category.

(18) $$\mathrm{mAP}=\frac{1}{N}\sum_{t=1}^{N}{\mathrm{AP}}_{i}$$

In Equation (18), $AP_{i}$ denotes the average precision value for class index *i*, and *N* represents the total number of classes in the training dataset. Setting the intersection over union (IoU) threshold to 0.5 results in the average precision being referred to as mAP@0.5. Conversely, when the IoU value ranges from 0.5 to 0.95, the average precision is denoted as mAP@0.5:0.95.

### 3.3. Optimal Decision-Making Experiments for SimAM Addition

This experiment assessed the impact of integrating the SimAM attention mechanism into the YOLOv8 architecture with SENetV2 and BiFPN frameworks. SimAM (Simple Attention Module) is a lightweight channel attention mechanism that enhances feature representation without adding extra convolution operations. In the experiments, SimAM was combined with SENetV2 to form the SESA module, incorporated into both the backbone and head layers, to evaluate its impact on model performance. The results show that integrating SimAM with SENetV2 (SESA) increased mAP@0.5 to 86.3%, significantly outperforming the original YOLOv8n model. This improvement is mainly due to SimAM’s ability to adaptively highlight important channel features during feature extraction while suppressing irrelevant ones. As a parameter-free method, SimAM avoids additional convolution operations, leading to no significant increase in inference time while enhancing accuracy.

The study also analyzed the effects of incorporating SimAM into various detection layers: small-object, large-object, and neck layers. The detection outcomes of incorporating SimAM at different positions are summarized in Table 2. The results show that applying SimAM only to the small-object layer achieved an mAP@0.5 of 83.7%, indicating some benefit in small-object detection but not fully optimizing the feature representation. When applied to the large-object layer, the mAP@0.5 remained the same, suggesting minimal improvement for large objects, which already have robust feature representations. Applying SimAM to the neck layer resulted in a decrease in mAP@0.5 to 82.5%, likely due to redundant attention computations across layers, leading to excessive filtering and reduced performance. The best results were achieved by incorporating SimAM into the backbone layer, which enhanced detection accuracy with minimal computational cost. This approach is particularly effective for multi-scale, multi-object detection in marine environments, where adaptability is essential.

### 3.4. Ablation Experiments

The proposed enhancements—the SESA fusion attention mechanism, BiFPN, and Soft-NMS—were validated via ablation experiments, illustrating their substantial impact in terms of improving the performance of the baseline YOLOv8 model. The “✓” symbol denotes the application of a specific evaluation method. Table 3 indicates that YOLOv8-n functions as the baseline for these experiments. The findings indicate that each enhancement significantly influences detection accuracy, recall, mAP@0.5, mAP@0.5:0.95, FLOPs, and model parameters, with especially marked improvements in the mAP metrics.

The introduction of the SESA module markedly improved the feature representation capability of Model B. The SESA module enhances the model’s sensitivity to essential features while mitigating redundancy via an inter-channel information recalibration mechanism. The mAP@0.5 improved from 82.4% to 84.2%, while mAP@0.5:0.95 increased to 56.8%. The number of model parametersincreased to 3,028,792, while the FLOPs remained at 8.2 G, demonstrating that SESA significantly enhances detection accuracy without additional computational costs.

The BiFPN module’s introduction enhanced Model C’s performance in multi-scale feature fusion, particularly improving small object detection. This module enhances feature integration via efficient bidirectional pathways and an adaptive weight allocation mechanism, achieving an mAP@0.5 of 84.1% and an mAP@0.5:0.95 of 54.9%. The number of parameters was reduced to 2,789,572, while the computational load remained at 8.2 G, illustrating the module’s capacity to improve performance without compromising with respect to a lightweight model structure.

The integration of Soft-NMS into Model D substantially reduced false detections associated with traditional NMS in high-overlap situations. The adjustment resulted in a significant enhancement in performance, with the mAP@0.5 increasing to 85.0% and mAP@0.5:0.95 rising to 57.6%. The increase in FLOPs to 8.9G was accompanied by a notable enhancement in detection performance in dense scenes due to the implementation of this post-processing strategy, resulting in an effective balance between accuracy and efficiency.

The incorporation of Soft-NMS into Model D significantly diminished false detections linked to conventional NMS in scenarios with high overlap. The adjustment led to a notable improvement in performance, with the mAP@0.5 increasing to 85.0% and mAP@0.5:0.95 rising to 57.6%. The increase in FLOPs to 8.9 G resulted in a significant improvement in detection performance in dense scenes, which is attributable to the application of this post-processing strategy, thereby achieving a balance between accuracy and efficiency.

### 3.5. Comparison of the Overall Performance of YOLO-SEA and YOLOv8n

Table 4 presents a detailed performance comparison between the YOLOv8n model and the YOLO-SEA model introduced in this study. The enhanced algorithm demonstrates superior performance compared to YOLOv8 in terms of average mAP accuracy, achieving improvements of 5.4% in mAP@0.5 and 7.2% in mAP@0.5:0.95 for YOLO-SEA. The enhanced model algorithm exhibits marginally reduced parameters and lighter weights in terms of model space complexity, while the FLOPs remain consistent with those of the original model. The enhanced model demonstrates increased accuracy with a comparatively low parameter count while maintaining real-time inference speed. The denser fusion paths, broader receptive fields, and enhanced contextual information offer adequate feature support for detection processes, thereby improving target localization and classification, particularly in complex background scenarios.

**Figure 8:**
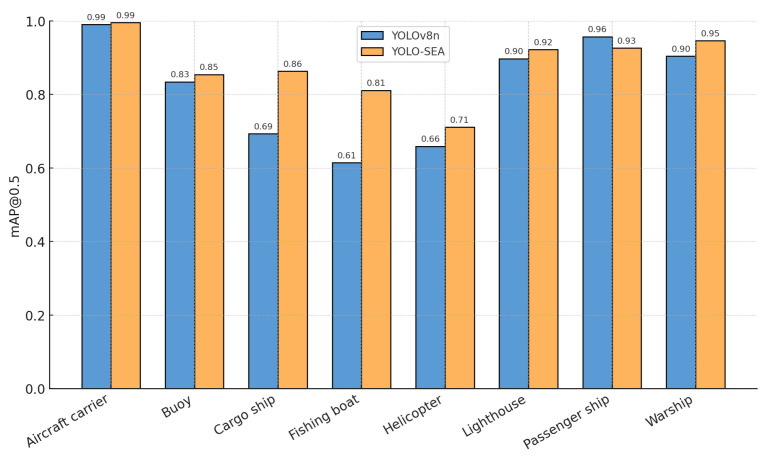
Performance analysis of 8 categories in the dataset.

Figure 8 presents the mAP@0.5 for eight classes within the test set, contrasting YOLO-SEA with YOLOv8n. The proposed model demonstrates improvements across all categories, with the most notable enhancements in the cargo ship and fishing boat classes. The experimental results indicate that the enhanced model produces detection regions that better correspond to the actual object boundaries, improving the accuracy of multi-class object detection and localization in maritime environments.

**Figure 9:**
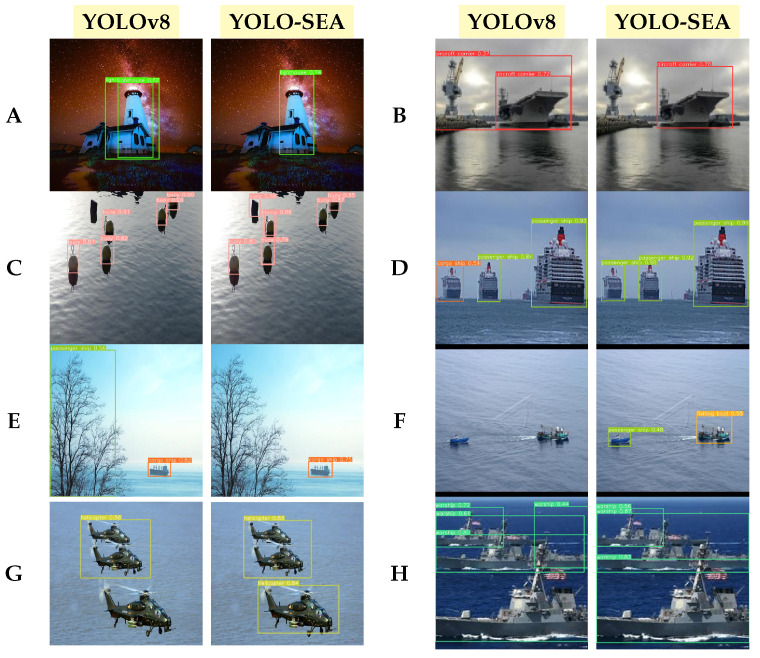
Detection results for various maritime objects in the test set. Labels (**A**–**H**) correspond to: (**A**), lighthouse; (**B**), aircraft carrier; (**C**), buoy; (**D**), passenger ship; (**E**), cargo ship; (**F**), fishing boat; (**G**), helicopter; (**H**), warship.

This paper visualizes and analyzes the detection results to evaluate the effectiveness of the YOLO-SEA model in identifying various categories of objects at sea. Figure 9 presents a comparative analysis of the detection performance between the YOLO-SEA model and the original YOLOv8 across various maritime scenarios. Analysis of various scenarios indicates that YOLO-SEA surpasses YOLOv8 in target differentiation, false detection management, omission correction, and the detection of small targets against complex backgrounds. In scenario (A), YOLOv8 misclassifies a house as a lighthouse, achieving a recognition accuracy of 62%. In contrast, YOLO-SEA effectively eliminates interfering targets and accurately identifies a lighthouse with a recognition accuracy of 74%. This demonstrates YOLO-SEA’s superior classification capability in complex backgrounds compared to the original YOLOv8.

In scene (B), YOLOv8 mistakenly identifies other objects as aircraft carriers, with a recognition accuracy of 72%, but false detections persist. YOLO-SEA improves recognition accuracy to 76%, successfully identifying the aircraft carrier and demonstrating its stronger target feature extraction. Scene (C) showcases YOLO-SEA’s advantage in detecting small targets. While YOLOv8 misses the buoys, YOLO-SEA correctly detects all of them, proving its robustness in small object detection. In scene (D), YOLOv8 misidentifies a passenger ship as a cargo ship, but YOLO-SEA accurately classifies the vessel, achieving over 90% accuracy and showcasing better stability in distinguishing similar targets.

Scene (E) reveals that YOLOv8, though accurate with cargo ships, mistakenly classifies an adjacent tree, causing detection ambiguity. YOLO-SEA maintains correct cargo ship recognition while improving target boundary precision. Scene (F) highlights YOLO-SEA’s capability to detect small targets at a distance, whereas YOLOv8 fails to spot a fishing boat. YOLO-SEA excels in detecting low-resolution features in complex scenes. Scene (G) emphasizes YOLO-SEA’s multi-target detection strength, outperforming YOLOv8, which fails to detect a helicopter in a multi-target setting. YOLO-SEA achieves over 80% accuracy. In scene (H), YOLOv8 struggles with redundant detection frames for warships, whereas YOLO-SEA eliminates redundancy and boosts recognition accuracy. Overall, YOLO-SEA, enhanced by improved modules, excels in maritime object detection, providing an efficient solution for complex surveillance tasks.

**Figure 10:**
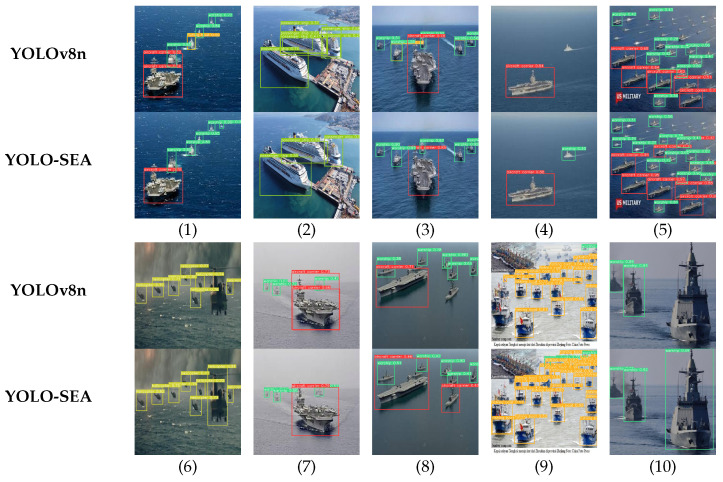
Visualization comparison of detection results by YOLOv8n and YOLO-SEA across ten maritime scenes.

The multi-target detection task requires the model to identify and localize multiple targets simultaneously, with the complex maritime environment adding to the detection challenges. Experimental results show that YOLOv8 suffers from issues like leakage detection, false detections, and overlapping anchor frames in multi-target scenarios. When targets are close or the background is cluttered, some targets are missed, leading to leakage detection. Additionally, similar targets are often misclassified, reducing accuracy. YOLOv8 also struggles with multiple detection frames for a single target, complicating processing. As shown in Figure 10, YOLO-SEA improves target differentiation and reduces misdetections by optimizing feature extraction and fusion, while Soft-NMS removes redundant frames, enhancing detection accuracy and stability. Key improvements include a shallow information-dense fusion module for better small target feature learning and multi-scale feature fusion to handle varying target sizes. This optimization boosts target differentiation in complex backgrounds, improving detection robustness. YOLO-SEA outperforms YOLOv8 in detecting small targets, managing multi-target occlusions, and adapting to complex environments, providing a more stable and efficient solution for maritime multi-target detection.

### 3.6. Evaluation of the Model’s Performance Across Various Weather Conditions

The marine environment is intricate and variable, presenting significant challenges for the accurate detection of ships, buoys, and other marine objects under adverse weather conditions. Obtaining a substantial quantity of high-quality images in adverse weather conditions within the actual marine environment presents significant challenges. This paper employs an artificial synthesis method to generate images of maritime objects across various weather conditions to evaluate the detection model’s performance. This paper synthesizes three typical severe weather scenarios—rain, fog, and low light—to create a hybrid weather test set. The YOLO-SEA model is subsequently evaluated using the test set to systematically analyze the impact of various severe weather conditions on maritime object detection performance. The experimental results elucidate the model’s adaptability in complex marine environments and serve as a reference for enhancing the robustness of maritime object detection. This content provides a detailed description of the image synthesis process across various weather conditions.

**Figure 11:**
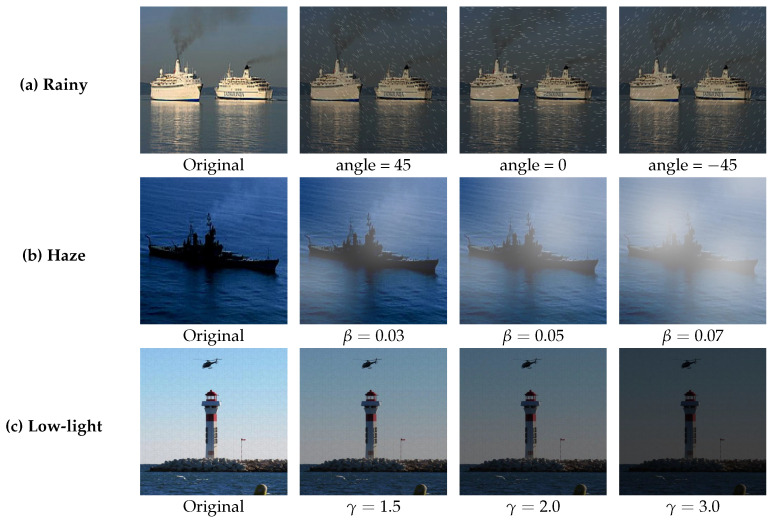
Visual representations of various objects subjected to extreme weather conditions at sea. Images depicting rainy weather: the original image alongside modified versions featuring rain bar angles of 45°, 0°, and −45°. Haze weather images: original image and images exhibiting haze concentrations of 0.03, 0.05, and 0.07. Low-light images: original image alongside images adjusted with gamma coefficients of 1.5, 2.0, and 3.0.

1. Synthesis of rain images: The rain layer exhibits various tilt angles. We integrate rainfall patterns with tilt angles of −45 degrees, 0 degrees, and 45 degrees into the preprocessed images of maritime objects to synthesize ship images under rainy conditions. The equation for synthesized rain is expressed as follows:
  (19) A(x)=αN(x)+(1−α)M(x)
  where N(x) represents the initial input image, M(x) denotes the generated simulated raindrop effect image, and α is the blending weight that regulates the impact of the raindrop effect on the final image. Typically, α assumes values between 0 and 1, with lower values indicating a more pronounced raindrop effect. This study establishes α=0.6 to equilibrate the original image with the raindrop effect, thereby aligning the simulation outcomes more closely with actual rainy-day conditions. Figure 11a presents an example of a synthesized image depicting a rainy day.
2. Synthesis of haze images: Haze is a prevalent adverse weather phenomenon encountered in maritime transportation. The atmospheric scattering theory posits that haze primarily results from the scattering of particles present in the atmosphere. Haze significantly diminishes image quality in the process of detecting objects at sea. To accurately replicate real sea conditions, the atmospheric scattering model [38] is employed to synthesize the haze image. Synthesized fog is expressed as follows:
  (20) I(x)=J(x)t(x)+A(x)(1−t(x))
  In this formula, I(x) denotes the synthesized dense haze image, J(x) signifies the original image, t(x) indicates medium transmittance, and A(x) represents global atmospheric light. The dielectric transmittance (t(x)) exhibits an exponential decay concerning the propagation distance of light, as described by $t\left(x\right)=e^{-\beta d(x)}$, where β represents the scattering factor attributed to atmospheric particles and d(x) denotes the distance to the target object. Adjusting the atmospheric scattering factor (β) allows for the generation of varying degrees of haze in images of maritime objects. In this experiment, β takes values of {0.03,0.05,0.07}. Figure 11b presents an example of a synthesized haze image.
3. The synthesis of low-light images indicates that low-light conditions at sea can cause blurring of a ship’s silhouette, potentially leading to confusion with coastal buildings and complicating detection efforts. Retinex theory posits that an image can be represented as the product of light and reflectivity. The formula is presented as follows:
  (21) S(x)=R(X)L(x)
  In this context, x∈Ω, where Ω represents the image domain, and the low-light image (S(x)) is derived by multiplying the reflectance image (R(x)) by the illumination image (L(x)). A low-light environment diminishes brightness and contrast and results in a loss of detail. This paper employs the gamma transform ($F\left(x\right)=X^{\gamma }$) to simulate low-light conditions, with γ representing the attenuation coefficient that regulates the extent of brightness attenuation. γ={1.5,2.0,3.0}. Figure 11c presents an example of a synthesized low-light image.

This experiment employs YOLOv8n as a baseline comparison model, training it on original natural images and testing it with mixed-weather images to evaluate the impact of severe weather conditions on detection performance for maritime objects. Table 5 illustrates that the detection performance on mixed severe-weather test images is notably diminished, with the original YOLOv8 model underperforming compared to our proposed YOLO-SEA model across multiple evaluation metrics. The YOLO-SEA model demonstrates the ability to mitigate specific environmental disturbances in mixed severe-weather test images, achieving enhanced ship detection accuracy while reducing the model’s parameter count relative to the original YOLOv8 model.

**Figure 12:**
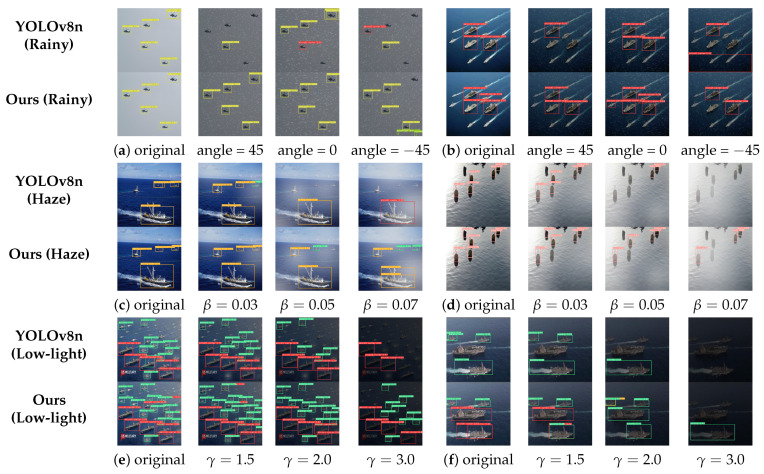
Detection examples of maritime objects under different weather conditions. Rows show detection results of YOLOv8n and our method under rainy, haze, and low-light conditions. Variations include changes in rain bar angle, haze concentration (β), and gamma correction (γ).

To evaluate the detection performance of the models across various environments, we present examples of maritime object detection under differing weather conditions for each model. This analysis assesses the relevance of the models in practical maritime contexts. Figure 12 presents the detection outcomes of YOLOv8n and the YOLO-SEA model introduced in this study across various weather conditions, encompassing both the original images and the detection results influenced by environmental factors.

Figure 12a,b illustrate that images before and following processing exhibit minimal impact on larger targets during moderate rainfall, whereas smaller targets are more susceptible to disturbances caused by raindrops from various angles. Our model demonstrates superior performance in minimizing leakage and false detection compared to the original YOLOv8 model, resulting in a notable enhancement in overall detection efficacy despite the persistence of some leakage instances. Figure 12c,d illustrate that varying concentrations of haze impact ship detection differently. In conditions of thin haze, the false detection rate is elevated (as observed in scene (c)). Conversely, as haze concentration increases, detection accuracy declines, particularly resulting in the omission of small target ships due to haze occlusion. The original YOLOv8 model demonstrates significant limitations in detecting targets under haze conditions, with β=0.07, whereas the YOLO-SEA model retains the ability to recognize target objects in high-haze environments. Despite a lower confidence level, YOLO-SEA offers more precise target location information.

The impact of varying lighting conditions on ship detection is notably pronounced in low-light environments. Figure 12e,f illustrate that an increase in the low-light parameter correlates with a decrease in image contrast, leading to less distinct ship features. This reduction in prominence adversely impacts the model’s capacity to identify detailed features, thereby compromising detection outcomes. At γ=3.0, the original YOLOv8 model demonstrates significant limitations in object detection, whereas the YOLO-SEA model maintains effective target identification. Furthermore, the localization capability of YOLO-SEA surpasses that of YOLOv8, even though an increase in γ results in a reduction in detections and an elevated risk of misdetection. In comparison to the original YOLOv8 model, YOLO-SEA exhibits enhanced robustness in detecting ships across various complex environments. Compared to the original YOLOv8 model, YOLO-SEA demonstrates enhanced robustness and improved accuracy in recognizing ship categories and positional information, as evidenced by both quantitative results and detection examples.

### 3.7. Comparison of the Performance of Leading Algorithms

This paper conducts a comprehensive evaluation of the detection performance of the YOLO-SEA model by comparing it with Faster R-CNN, SSD, RT-DETR, and various models from the YOLO series. As shown in Table 6, the tabular data indicates that YOLO-SEA demonstrates superior performance compared to existing models regarding detection accuracy (mAP@0.5 and mAP@0.5:0.95), particularly in the detection of small targets and complex backgrounds. YOLO-SEA achieves a favorable balance between the number of parameters and computational effort, enhancing its potential for practical applications, particularly in resource-intensive scenarios. There remain areas of concern, particularly regarding the optimization of lightweight design and computational efficiency, which could facilitate future improvements.

Conventional target detection models Faster R-CNN and SSD exhibit detection accuracies of 78.6 and 80.8, respectively—significantly lower than that of YOLO-SEA. Their reliance on Region Proposal Network (RPN) or Anchor-Based methods renders them susceptible to interference in complex backgrounds and results in substantial computational demands, complicating the fulfillment of real-time detection requirements. RT-DETR utilizes the Transformer architecture, known for its effective global feature extraction. However, its parameter count of 32 million and its 103.5 billion FLOPs result in significant computational overhead, surpassing the requirements for real-time applications. In contrast, YOLO-SEA employs only 2.8 million parameters and 8.1 billion FLOPs, resulting in a substantial reduction in computational resource consumption while achieving enhanced detection accuracy, particularly for small targets and complex backgrounds, demonstrating clear advantages.

The YOLO-series models are widely used for real-time object detection due to their lightweight design and high efficiency. Among them, YOLOv7 achieved a mAP@0.5 of 83.1, while YOLOv6 obtained a mAP@0.5:0.95 of 54.5. However, these gains came with increased computational complexity. For instance, YOLOv5 has a computational load of 16.0 GFLOPs, whereas YOLOv7 reaches 105.2 GFLOPs. Despite improved accuracy, their high computational cost limits their use in resource-constrained environments. YOLOv8, with a reduced count of 8.2 GFLOPs, still lags behind YOLO-SEA in detection accuracy. YOLO-SEA improves feature extraction through the SESA fusion attention module and uses BiFPN for effective feature fusion, achieving superior performance within the same computational limits.

Models YOLOv9 to YOLOv12 focused on reducing model size and complexity. While YOLOv9 showed a reduction in parameters and computational load, it still fell short of YOLO-SEA by 2.9 and 2.7 points in mAP@0.5 and mAP@0.5:0.95, respectively, indicating that lightweight optimizations can compromise accuracy. In contrast, YOLO-SEA strikes a better balance. YOLOv10 and YOLOv11 improved detection accuracy but still did not surpass YOLO-SEA, despite similar computational complexity, demonstrating YOLO-SEA’s superior feature representation. Although YOLOv11 and YOLOv12 offered slight improvements in the number of parameters and FLOPs, they still did not exceed the performance of YOLO-SEA, which offers an optimal balance between accuracy and efficiency.

YOLO-SEA stands out for balancing detection accuracy, computational efficiency, and model size, excelling particularly in small object detection and complex backgrounds. Compared to other YOLO models, YOLO-SEA significantly reduces computational resource consumption while outperforming previous versions, especially in the mAP@0.5:0.95 metric, with a notable score of 60.8. While it lags behind YOLOv9, YOLOv11, and YOLOv12 in terms of lightweight design, this gap presents opportunities for future optimization. Techniques such as pruning and knowledge distillation could further reduce the model size while maintaining precision, enhancing YOLO-SEA’s competitiveness in real-world applications.

### 3.8. Experiments on Generalizability

To evaluate the generalization ability and effectiveness of the YOLO-SEA model in maritime object detection, comparative experiments were conducted using the publicly available *6_class_final* [39] dataset. Two commonly utilized metrics, mAP@0.5 and mAP@0.5:0.95, were chosen to evaluate detection performance. As shown in Table 7, YOLO-SEA attained an mAP@0.5 of 81.7% and an mAP@0.5:0.95 of 61.6%, indicating a notable enhancement compared to the baseline YOLOv8n, which recorded an mAP@0.5 of 78.8% and an mAP@0.5:0.95 of 58.4%. This enhancement demonstrates that the proposed model exhibits superior performance in detecting objects across different scales, providing more accurate feature extraction while sustaining high detection accuracy in intricate maritime environments. It exhibited enhanced adaptability in identifying small objects and medium-to-large vessels. In the *Fish-b* (torpedo boat) and *Warship* categories, YOLO-SEA attained detection accuracies of 90.5% and 84.8%, respectively, exceeding YOLOv8n’s performance (89.7% and 77.2%) and surpassing all other models in the *Warship* category, thereby demonstrating its enhanced feature extraction capabilities. Furthermore, in the *Container* and *Cruise* categories, YOLO-SEA achieved detection accuracies of 71.3% and 97.2%, respectively, demonstrating significant enhancements compared to YOLOv8n, which recorded accuracies of 58.1% and 95.3%. It notably surpassed all YOLO-series and RT-DETR models in container detection, demonstrating enhanced generalization ability in identifying complex ship categories. YOLO-SEA achieved overall mAP@0.5 and mAP@0.5:0.95 scores of 81.7% and 61.6%, respectively, demonstrating superior performance compared to all other evaluated models, thereby confirming its effective detection capability in maritime contexts.

While YOLO-SEA excels in numerous categories, opportunities for optimization remain within specific areas. In category A, the mAP of YOLO-SEA is 51.6%, showing a minor improvement over YOLOv8n, at 50.6%, yet remaining below YOLOv11, which stands at 55.0%. This phenomenon may be associated with the smaller size and greater environmental disturbance of the targets in this category, which limits the model’s ability to learn their features. The RT-DETR series of models demonstrates superior performance in certain categories, such as Cruisers, with RT-DETR-l achieving 99.3% accuracy compared to YOLO-SEA’s 99.1%. This indicates that the RT-DETR models remain competitive in specific large target categories. In the future, YOLO-SEA can be optimized to improve its detection capabilities in complex environments with small targets, and its adaptability for specific categories can be enhanced to further elevate its overall detection performance. YOLO-SEA demonstrates a strong generalization ability in marine environments, effectively adapting to various target detection tasks. It achieves high detection accuracy while remaining lightweight, offering a reliable solution for maritime surveillance, safety, and intelligent shipping.

## 4. Conclusions

This paper proposes YOLO-SEA, a high-precision, multi-class maritime object detection algorithm optimized for multi-target, multi-scale, and multi-category detection, particularly in complex marine environments. The model incorporates the SESA fusion attention mechanism within the backbone network, effectively integrating the advantages of both approaches in feature modeling. SENetV2 enhances global and contextual information by utilizing adaptive channel weights, whereas SimAM effectively captures essential features across both spatial and channel dimensions. The complementary synergy of the two enhances SESA’s ability to perceive key information while effectively suppressing redundant features, thereby significantly improving feature expression capability and detection accuracy.

The weighted bidirectional feature pyramid network (BiFPN) is employed to enhance feature fusion. The structure of the target object is tailored to align with maritime scenarios, thereby improving the detection capability for small targets. Additionally, target localization is refined using Soft-NMS to increase recall rates and detection stability. Experiments indicate that YOLO-SEA achieves a detection accuracy of mAP@0.5 = 88.2% on the dataset utilized in this study, surpassing that of the original model, YOLOv8-n, while also demonstrating consistent detection performance across various severe weather conditions. Furthermore, generalizability experiments conducted on various ocean-related datasets indicate that YOLO-SEA surpasses current mainstream methods regarding detection accuracy and computational efficiency, thereby exhibiting enhanced robustness and adaptability. YOLO-SEA effectively balances model compression and detection performance, making it appropriate for offshore mobile devices with constrained computational resources. It offers efficient and reliable detection solutions for ship monitoring, maritime cruising, and emergency rescue scenarios. Future work will focus on the design of a more lightweight model, the improvement of small object detection accuracy, and further validation and optimization of the model’s performance under more complex maritime environments to broaden its practical applicability. In addition, we plan to expand the dataset to include additional categories such as engineering ships and offshore vessels, thereby enhancing the model’s ability to recognize a wider variety of maritime targets and improving its adaptability in diverse real-world scenarios.

## Figures and Tables

**Table 1:** Dataset details.

Type of Detection Targets	Description	Image Style
Small target	The target range is limited and easily overlooked.	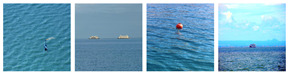
Large target	The target is more than half of the picture area.	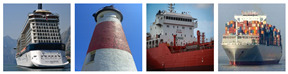
Target with complex background	Confusion of targets and complex backgrounds.	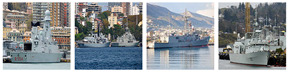
Multiple detection targets	Multiple detection targets are easy to miss.	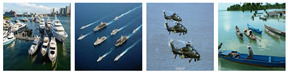
Detection targets at dusk	The target is unclear in the sunset.	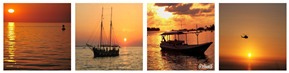
Detection target at night	The target is affected by light at night.	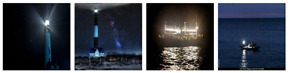

**Table 2:** Detection results of SimAM at various locations. A = SENetV2; B = BiFPN. (1) Small-target layer; (2) large-target layer; (3) neck; (4) backbone after fusion with A.

Model	mAP@0.5/%	Params/M	FLOPs/G
YOLOv8n+A+B (baseline)	82.8	2.81	8.3
YOLOv8n+A+B+SimAM (1)	83.7	2.80	8.1
YOLOv8n+A+B+SimAM (2)	83.7	2.80	8.1
YOLOv8n+A+B+SimAM (3)	82.5	2.80	8.1
YOLOv8n+SESA+B (4)	86.3	2.80	8.1

**Table 3:** Ablation study of the proposed method.

No.	SESA	BiFPN	Soft-NMS	mAP@0.5/%	mAP@0.5:0.95/%	Params/M	FLOPs/G
A				82.4	53.6	3.01	8.2
B	✓			84.2	56.8	3.03	8.2
C		✓		84.1	54.9	2.79	8.2
D			✓	85.0	57.6	3.01	8.9
E	✓	✓		86.3	56.4	2.80	8.1
F	✓	✓	✓	88.2	60.8	2.80	8.1

**Table 4:** Comprehensive comparison of YOLOv8n and the proposed model.

Model	mAP@0.5/%	mAP@0.5:0.95/%	Params/M	FLOPs/G
YOLOv8n	82.4	53.6	3.01	8.2
Ours	88.2	60.8	2.80	8.1

**Table 5:** Detection results under severe weather conditions.

Model	P/%	R/%	mAP@0.5/%	mAP@0.5:0.95/%	Params
YOLOv8n	68.0	73.8	75.4	45.7	3,007,208
Ours	73.4	80.7	82.9	56.9	2,800,564

**Table 6:** Comparison of model complexity and performance on the sea target dataset.

Model	mAP@0.5/%	mAP@0.5:0.95/%	Params/M	FLOPs/G
Faster R-CNN	78.6	–	–	–
SSD	80.8	–	–	–
RT-DETR-L	68.2	42.0	32.0	103.5
RT-DETR-X	80.2	53.2	65.5	222.5
ResNet-50	81.0	55.3	42.0	125.7
ResNet-101	80.7	54.4	60.9	186.3
YOLOv5	82.8	53.7	7.0	16.0
YOLOv6	82.1	54.5	4.2	11.9
YOLOv7	83.1	53.5	37.2	105.2
YOLOv8	82.4	53.6	3.0	8.2
YOLOv9	85.3	58.1	2.0	7.6
YOLOv10	84.7	56.8	2.7	8.2
YOLOv11	85.6	59.0	2.6	6.3
YOLOv12	84.8	56.0	2.6	6.3
YOLO-SEA (Ours)	88.2	60.8	2.8	8.1

**Table 7:** Comparison of performance in sea target detection using the *6-class final* dataset.

Model	mAP@0.5	mAP@0.5:0.95	A	Buoy	Container	Cruise	Fish-b	Warship
Faster R-CNN	71.25	-	39.76	89.54	47.36	95.34	84.08	71.44
SSD	71.85	-	38.99	87.78	50.12	93.26	87.33	73.62
RT-DETR-l	79.70	59.2	50.80	99.30	59.80	96.50	90.40	81.20
RT-DETR-x	79.90	58.8	47.70	97.60	67.90	95.00	89.90	81.20
Resnet50	79.60	60.2	49.10	99.20	65.50	95.50	88.90	79.60
Resnet101	79.70	59.9	46.50	99.10	60.30	97.00	91.80	78.20
YOLOv5	79.10	56.9	57.80	98.00	52.60	97.40	88.60	80.40
YOLOv6	78.60	58.2	49.60	98.60	58.40	96.60	88.80	79.30
YOLOv7	75.10	51.6	49.40	97.60	48.90	93.90	82.20	78.40
YOLOv8	78.80	58.4	50.60	97.80	58.10	95.30	89.70	77.20
YOLOv9	79.60	60.6	49.20	99.30	57.40	98.50	90.20	83.10
YOLOv10	80.90	60.5	49.40	99.30	62.80	97.50	91.60	84.60
YOLOv11	80.30	60.4	55.00	97.90	61.10	97.90	91.40	78.20
YOLOv12	79.10	60.0	48.10	98.50	57.70	97.60	91.30	81.40
YOLO-SEA (Ours)	81.70	61.6	51.60	99.10	71.30	97.20	90.50	84.80

All values are expressed in percentage (%).

## Data Availability

All data included in this study are available upon request by contact with the corresponding author.
